# Evidence of avian-mediated long distance dispersal in American tardigrades

**DOI:** 10.7717/peerj.5035

**Published:** 2018-07-04

**Authors:** Matthew J. Mogle, Scott A. Kimball, William R. Miller, Richard D. McKown

**Affiliations:** 1 Department of Biology and Chemistry, Baker University, Baldwin City, KS, United States of America; 2 Mid America Parasitology Services, LLC, Juniata, NE, United States of America

**Keywords:** Bird nest, Migration, Transport, Ectozoochory, Dispersal, Plumage, Biogeography, Tardigrade

## Abstract

Terrestrial tardigrades, commonly known as “water bears”, are part of a phylum of microscopic, aquatic invertebrates famous for cryptobiosis and space travel, but little is known about their modes of dispersal on Earth. Wind is assumed, but not truly demonstrated, to be the major method of global dispersal. Yet, some water bear distribution patterns cannot be explained by patterns of prevailing winds. Mammals and birds have been proposed as potential animal vectors. Importantly, most nearctic-neotropical migrant birds move north and south, with many crossing the equator, whereas prevailing winds move west to east or east to west but do not cross the equator. When multiplied by billions of birds over tens of millions of years, if the ectozoochory of tardigrades by birds is true then both regional and intercontinental patterns can be better explained. To test for the potential role of birds in tardigrade dispersal, the nests of 10 species for birds were examined. Seventy percent of nests were positive for tardigrades, demonstrating that some birds are in a position for transference. The carcasses of eight birds (six species) found dead from window strikes and a Sandhill Crane (Grus canadensis) found dead during routine surveys were also examined. Of the birds examined, 66% yielded tardigrades from two classes, three orders, and five species, including juveniles, adults, and eggs, suggesting that many bird species are potential vectors for many species of tardigrades. Our data support the hypothesis of avian-mediated long distance dispersal of tardigrades and provide evidence that further investigation is warranted.

## Introduction

Terrestrial tardigrades are microscopic, aquatic invertebrates commonly found in the water trapped in the interstitial spaces in mosses and lichens. Tardigrades are famous for cryptobiosis and space travel (Miller, 1997; Jönsson et al., 2008). Tardigrade populations exist on every continent (McInnes, 1994; Meyer, 2013), but little is known about their modes of dispersal. In 1845, Hooker (1845) postulated that winds could act as a long distance dispersal (LDD) mechanism for invertebrates. Today, tardigradologists support the idea that tardigrade distribution is facilitated by winds (Kinchin, 1994) based on the default assumption that given a lack of evidence supporting other logical mechanisms for tardigrade dispersal, wind must be accepted (Ockham’s Razor).

Terrestrial tardigrades have been shown to have limited capacity for moving themselves over any distance. When they are active in moist habitat (moss or lichen), a condition that may exist for only a few hours a year, Shcherbakov et al. (2010) demonstrated that tardigrades move less than 23 mm/h. If tardigrades are incapable of moving easily through the landscape, even over short distances within a single unit of habitat, tardigrade habitats should show high levels of heterogeneity in community diversity. Miller, Miller & Heatwole (1994) demonstrated high variation of density and diversity within a single sample and Meyer (2006) showed differences between seemingly identical cryptogam habitats. These findings were supported by Nelson & Adkins (2001) who found that five species of tardigrade did not migrate relative to moisture conditions within a Tennessee moss. Collectively, these studies demonstrate that tardigrades do not disperse efficiently when relying on their own motility.

Although tardigrades seem incapable of moving themselves over long distances, the phylum is globally distributed (McInnes & Pugh, 1998; Pilato & Binda, 2001; McInnes & Pugh, 2007). Nevertheless, local and regional diversity and distribution data have many large voids, especially in the Americas (Meyer, 2013; Kaczmarek, Michalczyk & McInnes, 2014; Kaczmarek, Michalczyk & McInnes, 2015; Kaczmarek, Michalczyk & McInnes, 2016). In the absence of evidence to the contrary, the disjunct and widespread distribution of tardigrades, including tardigrade presence on remote land masses such as Antarctica (Dastych, 1984; Miller, Miller & Heatwole, 1996), Heard Island (Miller, McInnes & Bergstrom, 2005), Easter Island (Kristensen, Michalczyk & Kaczmarek, 2010) or even Hawaii (Binda & Pilato, 1994), has been explained as a consequence of global wind patterns.

Actual evidence of wind dispersal is scarce (Guil, 2011; McInnes, 2011). Nevertheless, Sudzuki (1972) showed the potential for wind speeds greater than 2 m/s to disperse tardigrades over short distances (≤5 m), Kristensen (1987) reported observing tardigrades trapped in rain drops on Greenland’s Disko Island, and Janiec (1996) demonstrated that moss, lichen and tardigrades could be dispersed short distances by winds. More recently, Munoz et al. (2004) modeled the circumpolar distribution of tardigrade habitat (moss propagules) around Antarctica and Nkem et al. (2006) caught tardigrades in open faced traps in the Dry Valleys of Antarctica. If tardigrades are dispersed by the winds, we should expect genus and species distribution to mimic the prevailing winds across a continent and between continents.

**Figure 1:**
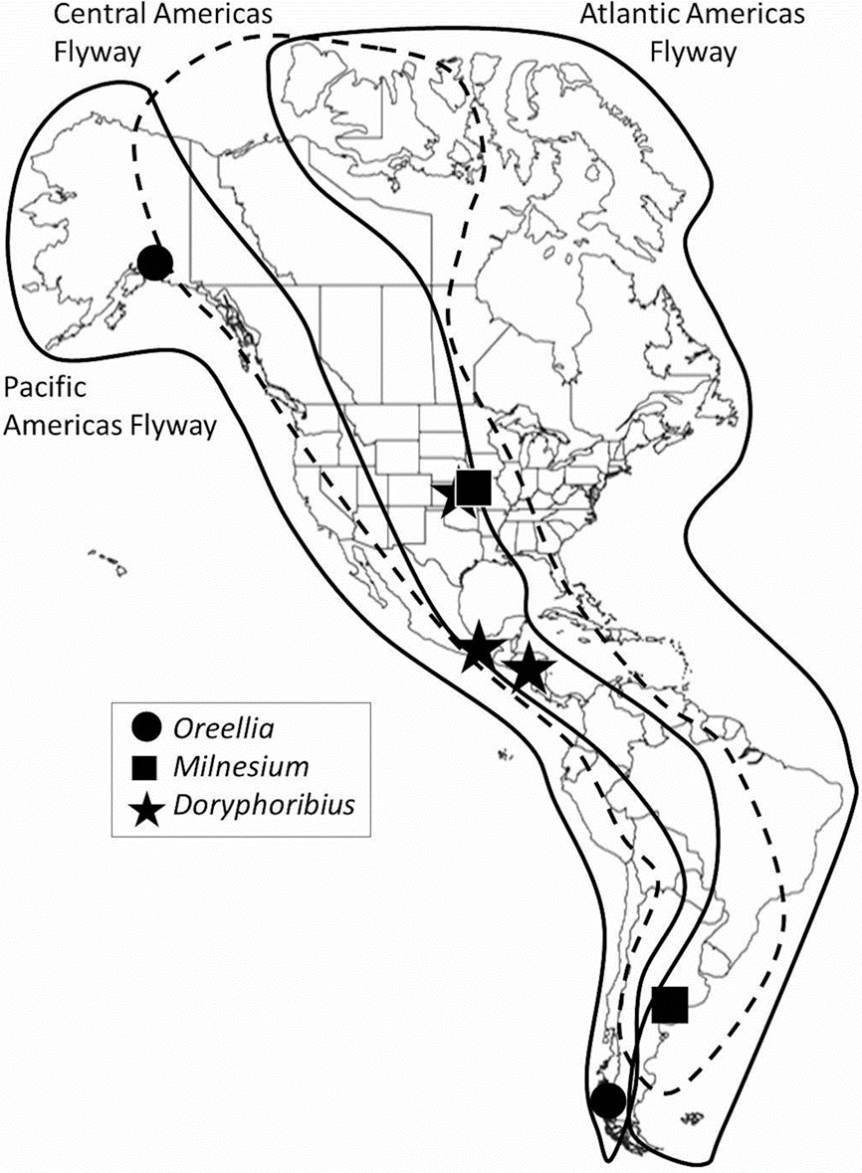
Migration flyways in the Americas, with examples of disjunct distributions of some tardigrades.

It is unlikely that local, regional or hemispherical biogeographic patterns of tardigrades are solely due to wind because the same tardigrade taxa have been found in areas not connected by Earth’s wind patterns. For example, the genus *Oreellia* has only two species: one known from the southern hemisphere, specifically Australia and Chile (Dastych, McInnes & Claxton, 1998), and the other from Alaska (Calloway et al., 2011) (Fig. 1). In 2013, *Doryphoribius dawkinsi* Michalcyzk & Kaczmarek, 2010 from Costa Rica was found 20 degrees of latitude north in the canopy of eastern Kansas forests (Spiers et al., 2013). Then Chappell et al. (2015) found *Doryphoribius gibber* Beasley & Pilato, 1987 also in the Kansas forest canopy where its only previous records have been Arkansas and southern Mexico (Pilato & Lisi, 2006). Most recently, *Milnesium beatae* Roszkowska, Ostrowska and Kaczmarek, 2015 was described from moss habitat on rocks in Argentina. A few months later, Tibbs, Emmanuels & Miller (2016) discovered the same species 9,000 km north in lichen habitat in the canopy of Red Oak trees in Kansas. These researchers have suggested migrating birds may serve as vectors due to their interactions with tardigrade habitat (mosses and lichens) during nest building and foraging activities. Chappell et al. (2015) suggested that bark hugging migratory species, such as the red-breasted nuthatch (*Sitta canadensis*), the brown creeper (*Certhia americana*) or the yellow-bellied sapsucker (*Sphyrapicus varius*), also interact with tardigrade habitat and in so doing may pick up active tardigrades, tardigrade eggs, or tuns among other bark detritus in their feathers.

Numerous examples of avian ectozoochory are cited in the literature. Aquatic examples are summarized by Coughlan et al. (2017), though examples of birds serving as agents of LDD in invertebrates are limited to relatively large propagules (>1 mm). However, Lewis et al. (2014) found evidence of trans-equatorial migrant birds carrying bryophyte diaspores in their plumage, suggesting that bird plumage has the ability to carry and transport propagules smaller in size than tardigrade tuns over long distances (>100 km). If bryophyte diaspores can be caught in bird plumage, we suggest that tardigrades could be picked up as well, providing an opportunity for avian-mediated ectozoochory of tardigrades.

Coughlan et al. (2017) outlined five requisite conditions for successful avian-mediated ectozoochory: (1) propagule contact with a vector, (2) propagule attachment to vector, (3) propagule survival during transport, (4) propagule detachment from the vector, and (5) suitable receiving environment for propagule establishment. Tardigrades may easily satisfy the third of these conditions, given the well-known cryptobiotic feats of many species (Miller, 1997). The fourth condition is likely, given the preening activity of birds and their propensity to molt feathers before, during, and/or after migration events (Rohwer, Butler & Froehlich, 2005). The last condition is likely as well, due to the high probability of a bird coming into contact with suitable habitat for tardigrades.

The purpose of this paper, then, is to demonstrate support for the hypothesis that birds facilitate tardigrade dispersal by demonstrating that the first and second of these conditions are met. We suggest that birds acquire tardigrades in their plumage through interactions with mosses and lichens as part of their nest-building and foraging activities. If birds are ectozoochorous agents of LDD in tardigrades, we predict that tardigrades will be found in bird nests and plumage.

## Materials and Methods

**Figure 2:**
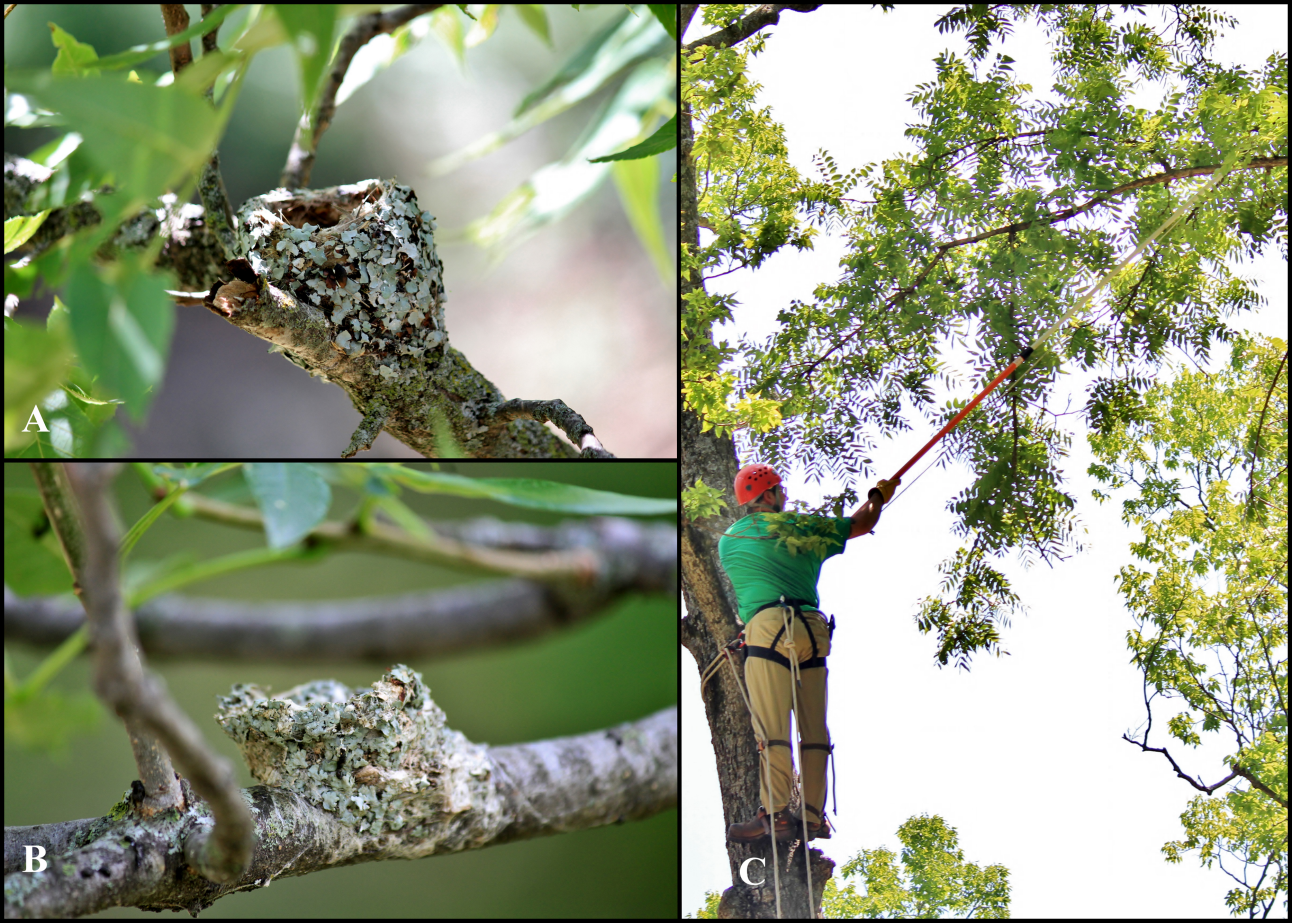
(A) Blue-gray gnatcatcher (*Polioptila caerulea*) nest covered with lichen in a black walnut (*Juglans nigra*) in Wabaunsee County, KS. (B) Ruby-throated hummingbird (*Archilochus colubris*) nest constructed with lichen in a green ash (*Fraxinus pennsylvanica*) in Douglas County, KS. C. SAK collecting the blue-gray gnatcatcher nest.

We collected entire bird nests opportunistically from late spring to mid-summer 2016 after young had fledged (Fig. 2). Each nest was treated as an independent sample. One half of each nest was soaked in approximately 100 mL of water overnight. The nest material was squeezed and removed from the sample and the debris was washed through a stacked series of soil particle separation sieves (400-200-100-50 µ). The debris in the bottom, finest mesh layer was back washed into a dish, allowed to settle, and examined for tardigrades. Then, three 1 mL aliquots were taken with a plastic pipette from each sample and examined under a dissecting scope at 25× and reflected light. This is the same processing technique used by Miller (1997) for tardigrades from cryptogams. Tardigrades found in nests were pooled for analysis.

In late spring 2016, we captured migratory and resident birds using mist nets and box traps near Baldwin City, KS, USA, as part of ongoing avian studies at Baker University. A sample of five to 15 contour feathers was pulled from the abdomen of each bird and stored in a sealed plastic bag (we presume that abdominal contour feathers are most likely to come into contact with tardigrade habitat). Each set of feathers was soaked in water for at least 3 hours in a small petri dish. Then, three 1 mL aliquots were taken with a plastic pipette from each sample and examined under a dissecting scope at 25× and reflected light (Miller, 1997).

We recovered birds that were recently dead from window strikes during late spring to mid-summer 2017. Each bird was treated as an independent sample. Processing of birds included several stages. First, the carcass was preserved in a plastic bag and frozen as soon as possible after discovery. Later, the bird was thawed, and its age and sex were determined. The bird carcass was placed in a glass jar with 100 mL of spring water and three to five drops of dish soap, after which the jar and its contents were agitated and left to soak for at least 3 hours. The carcass was removed and squeezed, and the debris remaining in the jar was washed through a stacked series of soil particle separation sieves (400-200-100-50 µ). The debris left in the bottom, finest mesh layer was back washed into a dish, allowed to settle, and examined. Twenty-five 1 mL aliquots were taken with a plastic pipette from each sample and examined under a dissecting scope at 25× and reflected light (Miller, 1997). All tardigrades from the birds were pooled for analysis.

Tardigrades were removed from samples using an Irwin loop (Schram & Davidson, 2012) and mounted on glass slides using polyvinyl alcohol (PVA) as the mounting medium (Salmon, 1951). Glass coverslips were sealed with fingernail polish, and the location of each tardigrade was marked by two small dots (left and right) with a permanent marker for later identification.

An Olympus BX60 Differential Interference Contrast (DIC) microscope (Tokyo, Japan) at 400–1,000× was used to identify tardigrades. Identification was based on Ramazzotti & Mauchi (1983) and Pilato & Binda (2010). Nomenclature was based on Guidetti & Bertolani (2005), Degma & Guidetti (2007) and Degma, Bertolani & Guidetti (2009–2016).

All tardigrades discovered and identified in this study were considered to be living animals. Living tardigrades exist in three conditions: cryptobiosis (the lack of activity caused by dehydration leading to the “tun” stage), asphyxia (the lack of activity caused by too much water leading to an animal becoming fully extended), and the active state (in wet conditions during which animals move about the habitat, eat, grow, and reproduce) (Ramazzotti & Mauchi, 1983). In the processing of tardigrades for study, we endeavor to push them into asphyxia by submerging the habitat sample in excess water, because in the fully extended condition they lose the ability to hold onto the habitat and float free, they are easier to see, and because asphyxiated tardigrades are best for mounting on slides with the least distortion and deformity. When a terrestrial tardigrade dies, it disintegrates quickly into unrecognizable detritus (Kinchin, 1994). Thus, all reported tardigrades in this and other studies were alive when recognized and mounted on a slide.

Trapping and collection of avian samples was conducted under US Federal Bird Banding Permit number #20076-B and KS Scientific, Education, or Exhibition Wildlife Permit numbers SC-052-2016 and SC-004-2017.

## Results

During the summer of 2016, nests from three different states and 10 different species of birds were collected and examined (Table 1). Seventy percent of bird nests examined yielded tardigrades. Seventy-four tardigrades were recovered from the seven nests. Two classes, three orders, seven genera, at least eight species were represented. In addition to mature tardigrades, eight eggs from four different species and specimens in simplex were found (Table 1).

**Table 1:** Density and diversity of tardigrades found in bird nests.

**Bird species:**	UNKN^a^	SAPH^b^	EABL^c^	EAPH^d^	CARW^e^	BRTH^f^	UNKN	BGGN^g^	RTHU^h^	AMRO^i^
State	KS	MT	KS	KS	KS	KS	MA	KS	KS	KS
Nest substrate	Grass	Unknown	Nest Box	Rock	Canoe	*Forsythia*	Ground	*Juglans nigra*	Unknown	*Pinus ponderosa*
Habitat visible	No	No	No	Moss	Both	Lichen	No	Lichen	Lichen	No
Density/nest	0	0	0	1	2	3	6	7	14	35
Diversity/nest	0	0	0	1	1	1	2	3	5	3
**Tardigrades found:**										
Heterotardigrada										
Echinsicidae										
*Echiniscus sp.*								4	1	
*Echiniscus maucci*									1	
Eutardigrada										
Apochela										
*Milnesium 1*				1						2
*Milnesium 2*							1			
*Milnesium 3*							1		1	7
*Milnesium 4*										1
Parachela										
*Hypsibius convergens*										22
*Ramazzottius sp.*									1	
*Macrobiotus hufelandi*						2				
*Macrobiotus sp.*										1
*Minibiotus sp.*							3			
*Paramacrobiotus tonolli*					1			1	5	
Simplex					1				1	1
Eggs						1		2	4	1

**Notes.**

a Unknown.

b Say’s phoebe (*Sayornis saya*).

c Eastern bluebird (*Sialia sialis*).

d Eastern phoebe (*Sayornis phoebe*).

e Carolina wren (*Thryothorus ludovicianus*).

f Brown thrasher (*Toxostoma rufum*).

g Blue-gray gnatcatcher (*Polioptila caerulea*).

h Ruby-throated hummingbird (*Archilochus colubris*).

i American robin (*Turdus migratorius*).

During the fall of 2016, feather samples from eight species (one individual of each species) of live birds were examined: white-throated sparrow (*Zonotrichia albicollis*), Swainson’s thrush (*Catharus ustulatus*), rose-breasted grosbeak (*Pheucticus ludovicianus*), red-bellied woodpecker (*Melanerpes carolinus*), tufted titmouse (*Baeolophus bicolor*), black-capped chickadee (*Poecile atricapillus*), northern cardinal (*Cardinalis cardinalis*), and brown-headed cowbird (*Molothrus ater*). No tardigrades were detected in these samples and none of the species of birds captured for feather examination overlapped with the species of bird nests examined.

During the summer of 2017, eight dead birds (six different species) were collected and examined in Kansas. Five of the eight specimens yielded seven tardigrades, which represented two orders, three genera, and four species. In addition to mature tardigrades, a juvenile and an identifiable egg were found on the Northern Flicker (*Colaptes auratus*) and on a White-throated Sparrow, respectively (Table 2). No tardigrades were found on the Mourning Dove (*Zenaida macroura*), a Northern Cardinal (*Cardinalis cardinalis*), or a House Finch (*Haemorhous mexicanus*), and consequently these specimens were not included in Table 2. In addition to specimens from Kansas, three unidentified tardigrades were found in plumage washings from a Sandhill Crane (*Grus canadensis*) recovered in Nebraska bringing the yield to six of nine birds (67%) positive for tardigrades.

**Table 2:** Tardigrades found on carcasses of recently-dead birds.

**Bird specimen:**	SACR^a^	WTSP^b^	WTSP	RTHU^c^	YSFL^d^	RWBL^e^
State	NE	KS	KS	KS	KS	MA
**Tardigrades Found:**						
Unknown	3					
Heterotardigrada						
Echinsicidae						
*Echiniscus* (adult)						1
*Echiniscus* (juvenile)					1	
Eutardigrada						
Apochela						
*Milnesium*		1				
Parachela						
*Diphascon*				1		
*Paramacrobiotus* (egg)			1			

**Notes.**

a Sandhill crane (*Grus canadensis*).

b White-throated sparrow (*Zonotrichia albicollis*).

c Ruby-throated hummingbird (*Archilochus colubris*).

d Northern flicker (*Colaptes auratus*).

e Red-winged blackbird (*Agelaius phoeniceus*).

## Discussion

Given the ubiquity of mosses and lichens in the landscape, many bird species have numerous opportunities to interact with tardigrade habitat. For example, several species, including the blue-gray gnatcatcher (*Polioptila caerulea*) and the ruby-throated hummingbird (*Archilochus colubris*), construct their nests from lichens and/or mosses (Fig. 2). Many of these same species are nearctic-neotropical migrants, moving between temperate and tropical latitudes annually, and as such may offer opportunities for long-distance tardigrade dispersal. In addition to gathering lichen to build their nests, these birds sit on their nests for extended periods of time while incubating eggs and caring for young. When it rains and the bird is on the nest, tardigrades in the nest material become active and may move onto the bird. As the water on the feathers dries, the tardigrades would form tuns and impurities in the water would form a biofilm that would impart adhesion for small particles such as cryptobiotic tardigrades.

The presence of tardigrades in bird nests, including adults, eggs, and individuals in simplex, demonstrates that the tardigrade populations living in nests have the potential to be healthy, reproducing, and growing. In addition, the presence of tardigrades in the nests of 70% of the bird species examined indicates that many birds consider tardigrade habitat as suitable nest material. Thus, many species of tardigrades and many species of birds are associated with each other, supporting the first condition (i.e., opportunity for contact) of successful ectozoochory proposed by Coughlan et al. (2017).

Despite the opportunistic nature and the small sample size of the collections, Coughlan et al.’s (2017) second condition of avian-mediated tardigrade transport (i.e., attachment of propagule to vector) was met by demonstrating the presence of tardigrades on six of nine (67%) bird carcasses. The density of tardigrades on birds was 1/10th the density of tardigrades found in bird nests suggesting a possible rate of transference from nest to bird plumage. Still, considering that billions of birds have undergone long distance migrations over tens of millions of years, even improbable events would occur frequently enough to establish populations in many disjunct locations.

## Conclusions

Avian-mediated LDD in tardigrades may help to protect tardigrade populations against local extirpations. Because tardigrades are incapable of relocating themselves even over short distances, populations may become vulnerable to more localized conditions and events. Consequently, the potential for populations to recover from local events may be facilitated through anemochory and ectozoochory, especially by birds. While wind is a likely mechanism of LDD in tardigrades, ectozoochory has the advantage that birds frequently encounter suitable tardigrade habitat and, consequently, are more likely to ensure successful dispersal than the more chaotic and unpredictable nature of anemochory.

Biogeographic patterns of tardigrade dispersion in the landscape should provide evidence of the relative contributions of wind and animal-mediated dispersal of this widespread group. However, tardigrade distribution data is lacking or absent altogether from much of the Earth, and consequently, studies of the biogeography and population biology of this phylum suffer from a lack of data. For example, Kaczmarek et al. (2016) suggested that current tardigrade distribution and diversity does not support the Great American Biodiversity Interchange (GABI) hypothesis. They concluded that the tardigrade faunas of North and South America are more similar to each other than either is to Central America. They stated that “high endemism in Central America suggests a more complex biogeographical process than predicted by the connection of two continents,” but recognized the spotty tardigrade biodiversity records of Nearctic and Neotropical regions.

Clearly, additional investigation into the local, regional, and global distribution of tardigrades is needed. Standard tools and techniques used to track dispersal of larger animals are not available to tardigrade researchers. Radio/GPS collars small enough to tag a tardigrade are still but a dream. The use of radioactive marking, release, and recapture of individuals at another site is in the realm of near impossibility. New developments in population genetics, however, may eventually give us the tools to investigate local, regional, and global dispersal. The use of metagenomics (Marco, 2011; Fierer et al., 2012; Velasco-Castrillon et al., 2015) to sequence and identify the DNA suite on a feather, nest, or sample shows promise. The ability to compare the genomes of morphospecies (Jørgensen, Møbjerg & Kristensen, 2007; Guil & Giribet, 2009) from different localities should provide clues to the evolution, taxonomy, distribution, and ecology of the phylum when a species-based library is available.

In summary, in addition to flying within local and regional environments, most birds fly between ecosystems and dozens of species migrate annually between and through North, Central, and South America. Thus, it appears that both migratory and resident birds have the ability and the opportunity to disperse tardigrades. Tardigrade dispersal by migratory birds is as plausible an explanation as wind dispersal for the widespread, but sometimes isolated populations of tardigrades. Birds have the potential to carry tardigrades across different habitats to new locations that are not connected by prevailing wind patterns. If these new locations are suitable, the transferred tardigrades can and will survive, grow, and reproduce.

## Additional Information and Declarations

### Data Availability

The following information was supplied regarding data availability: The raw data are included in the ‘Results’ section and Tables 1 and 2.
